# Repeated Tracheostomy Tube Cuff Rupture Due to Tracheobronchopathia Osteochondroplastica: A Case Report 

**Published:** 2015-09

**Authors:** Reza Nikandish, Mahammad Javad Fallahi, Beezhan Ziaian, Pooya Iranpour

**Affiliations:** 1 *Department of Anesthesia and Critical Care, Shiraz University of Medical Sciences, Shiraz, Iran. *; 2 *Department of Pulmonary and Critical Care, Shiraz University of Medical Sciences, Shiraz, Iran.*; 3 *Department of Cardiothoracic Surgery, Shiraz University of Medical Sciences, Shiraz, Iran.*; 4 *Department of Radiology, Shiraz University of Medical Sciences, Shiraz, Iran.*

**Keywords:** Intubation, Tracheobronchopathia osteochondroplastica, Tracheostomy

## Abstract

**Introduction:**

Tracheobronchopathia osteochondroplastica (TPO) is a rare benign disorder of the lower part of the trachea and the upper part of the main bronchi.

**Case Report:**

A case of tracheobronchopathia osteochondroplastica (TPO) diagnosed at the time of intubation in an intensive care unit due to difficulty when advancing the endotracheal tube beyond the vocal cords, is reported. A problem was encountered which had not been reported previously in TPO: repeated cuff rupture at the time of surgical tracheostomy occurred possibly because of bony and cartilaginous tissue located in the tracheal wall.

**Conclusion:**

In addition to difficulty of intubation, TPO may cause tracheostomy tube cuff rupture, which could be explained due to bonny calcification in the tracheal wall.

## Introduction

Tracheobronchopathia osteochondroplastica (TPO) is a rare benign disorder of the lower part of the trachea and the upper part of the main bronchi (1-3). Rokitansky first described TPO in 1855 (4).

The etiology, pathology, and natural history of TPO are unclear. Aschoff attributed it to a cartilaginous metaplasia of the sub epithelial connective tissue, which might well involve both calcification and ossification. It has also been suggested that it arises as a final evolutive stage of tracheobro -nchial amyloïdosis. Other hypotheses have been formulated: exostosis arising from the cartilaginous ring, local metabolic or inflammatory factors and infections, or chemical irritation. No direct relationship with either calcium or phosphorus metabolism has been established. Most patients affected with TPO are asymptomatic; and, rarely, the first presentation may be in the operating room during intubation, as has been presented previously in literature (5-8).

A case of TPO, with difficult intubation during airway management in an intensive care unit and with repeated rupture of the tracheostomy tube cuff at the time of surgical tracheostomy, is presented

## Case Report

A 67-year-old man was brought to emergency room with vertigo and ataxia 8 hours prior to admission. His head CT showed hypodencity in the left side of the medulla. His old chart showed that the basilar artery had 90 percent stenosis in a 4-vessel angiography. 

Following this, he has been scheduled for stent placement but it had been canceled because of the inability to intubate the patient after general anesthesia. He was subsequently referred to an optional ENT visit, which didn’t show any problem in indirect laryngoscopy, and a fibroptic evaluation of the lower airway was recommended. The following day, he developed dense right sided hemiplegia and respiratory distress and it was decided to intubate the patient; however, it was not possible to pass the 8.0 mm endotracheal tube (ETT) beyond the vocal cords. A smaller diameter tube (inner diameter, 6.5 mm) was placed in the larynx; however, the ETT was no able to advance but for a few centimeters from the vocal cords and it was fixed at 18 cm from the lips. The patient was transferred to an intensive care unit while he was conscious with right-sided weakness and had a massive air leak around the ETT during mechanical ventilation. 

**Fig.1:**
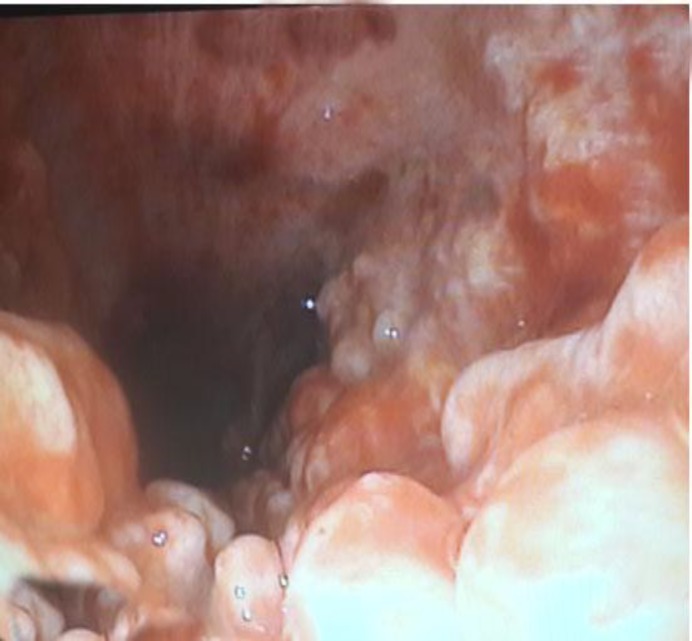
Tracheal mucosa as seen during the fibroptic bronchoscopy. Multiple red nodules protruding into the trachea spread over the anterior and lateral wall and sparing the posterior wall of the trachea

Direct laryngoscopy was performed and the ETT was changed to a larger one; however, it was not possible to advance the ETT deeper into the trachea. Fibroptic bronchoscopy (FOB) showed nodular mucosal hypertrophy, all over the trachea extending to the carina and main bronchi, which spared the membranous part; therefore, TPO was diagnosed bronchos- copically as the reason for difficult intubation (Fig.1). 

An Armored ETT (size 8) was passed into the trachea. Spiral chest CT showed that the trachea had very irregular borders (Fig’s.2,3).

**Fig 2:**
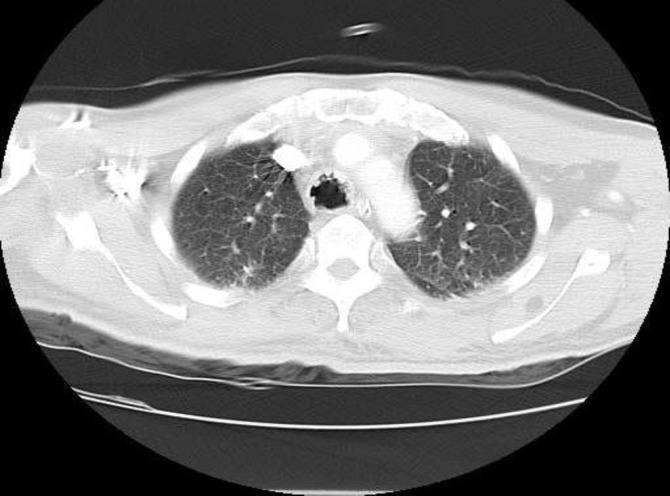
Axial contrast-enhanced CT image of trachea on lung windows showing nodular thickening of the tracheal wall

**Fig 3:**
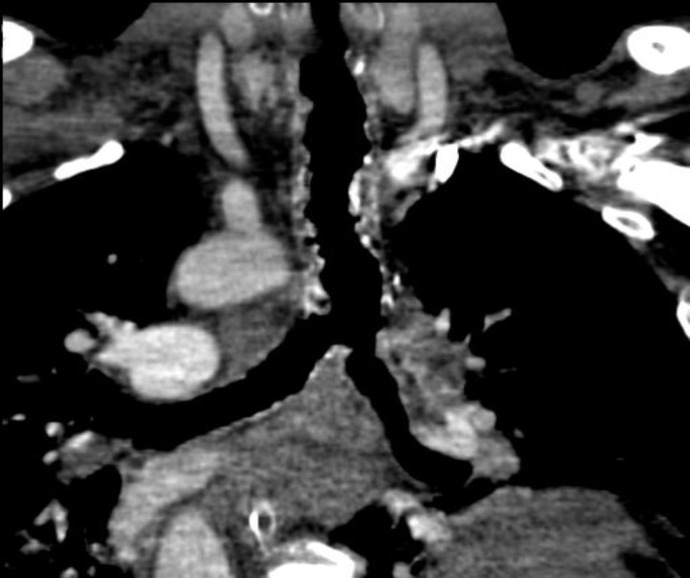
Coronal reconstructed CT scan reveals focal tracheal thickening and irregularity associated with multiple calcified nodules arising from inner wall

**Fig 4:**
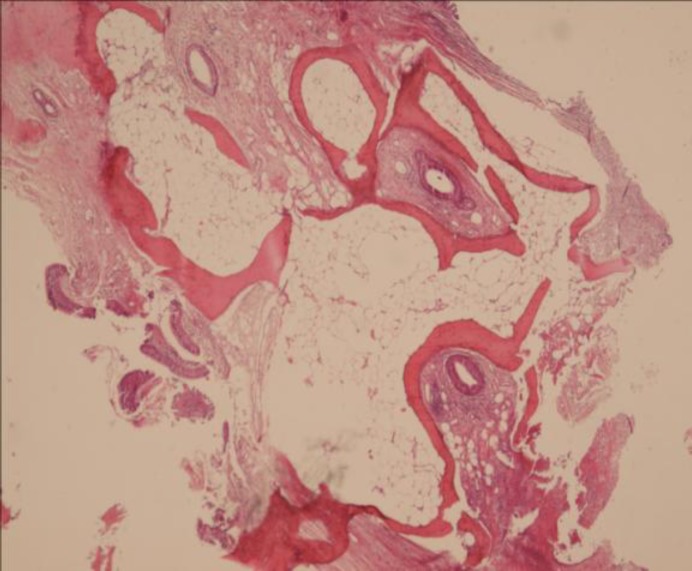
Sections from trachea shows fibrofatty tissue with bone formation (H&EX250)

In the following days he had a drop in his consciousness and repeat head CT showed more hypodencity in the pons and new hypodencities in the cerebellum. He was scheduled for surgical tracheostomy. During the open surgical tracheostomy, which was done by a thoracic surgeon, the trachea was stony hard and a tissue was sent for pathology from the tracheal wall, which showed fibrofatty tissue with bone formation (Fig.4).

 Another issue was repeated perforation of the tracheostomy tube cuff, which occured even after changing the tube 3 times during the tracheostomy procedure. There was a massive air leak around the tracheostomy tube after transferring the patient to ICU. Because of the inability to ventilate the patient, a laryngeal mask airway (LMA) was placed and the distal end and was clamped as a way to prevent massive air leak. Then the tracheostomy tube was removed and another one was passed into the trachea and this time the ETT cuff showed no perforation. 

## Discussion

TPO presented at the time of intubation has been reported previously during general anesthesia (4-7). The same scenario happened to this patient at the time of an optional neurointervention. It was not diagnosed due to a missed bronchoscopy follow up and finally presented difficult intubation during respiratory distress. As it is know, this is the second case of TPO diagnosed during the intensive care unit admission. Hantous et al, reported the first case of TPO, which was diagnosed during intensive care unit admission, in a 42-year-old man showing hypercapnic respiratory failure. It was detected bronchoscopically and was subsequently proven through biopsy. 

Another problem, which has not been reported previously, was repeated perforation of the tracheostomy tube cuff, which was encountered by the thoracic surgeon at the time of surgical tracheostomy. Perforation of cuff had occurred three times during tracheostomy tube insertion. Considering the fact that there was calcification and ossification observed during tissue biopsy, repeated rupture of the tracheostomy cuff could be explained by the trauma to the cuff due to the presence of bonny tissue in the tracheal wall. 

Due to large amounts of air leaks post tracheostomy, because of the damaged tracheostomy cuff, a classic laryngeal mask airway was used as a temporary measure to block the air leak through the mouth.

Use of the laryngeal mask to prevent massive air leak at the time of trachioplasty has been reported previously by Eckhardt et al. (8). 

## Conclusion

TPO may present itself for the first time during ICU admission and the tracheostomy cuff may be damaged by the bony growth in the tracheal wall.
